# Rapid automatic segmentation of abnormal tissue in late gadolinium enhancement cardiovascular magnetic resonance images for improved management of long-standing persistent atrial fibrillation

**DOI:** 10.1186/s12938-015-0083-8

**Published:** 2015-10-07

**Authors:** Archontis Giannakidis, Eva Nyktari, Jennifer Keegan, Iain Pierce, Irina Suman Horduna, Shouvik Haldar, Dudley J. Pennell, Raad Mohiaddin, Tom Wong, David N. Firmin

**Affiliations:** Cardiovascular Biomedical Research Unit, Royal Brompton Hospital, London, UK; National Heart and Lung Institute, Imperial College London, London, UK

**Keywords:** Late gadolinium enhancement, Cardiovascular magnetic resonance, Atrial fibrillation, Segmentation, Fibrosis, Ablation lesion, Three-dimensional visualization, Left atrium, Electro-anatomical mapping

## Abstract

**Background:**

Atrial fibrillation (AF) is the most common heart rhythm disorder. In order for late Gd enhancement cardiovascular magnetic resonance (LGE CMR) to ameliorate the AF management, the ready availability of the accurate enhancement segmentation is required. However, the computer-aided segmentation of enhancement in LGE CMR of AF is still an open question. Additionally, the number of centres that have reported successful application of LGE CMR to guide clinical AF strategies remains low, while the debate on LGE CMR’s diagnostic ability for AF still holds. The aim of this study is to propose a method that reliably distinguishes enhanced (abnormal) from non-enhanced (healthy) tissue within the left atrial wall of (pre-ablation and 3 months post-ablation) LGE CMR data-sets from long-standing persistent AF patients studied at our centre.

**Methods:**

Enhancement segmentation was achieved by employing thresholds benchmarked against the statistics of the whole left atrial blood-pool (LABP). The test-set cross-validation mechanism was applied to determine the input feature representation and algorithm that best predict enhancement threshold levels.

**Results:**

Global normalized intensity threshold levels *T*_*PRE*_ = 1 1/4 and *T*_*POST*_ = 1 5/8 were found to segment enhancement in data-sets acquired pre-ablation and at 3 months post-ablation, respectively. The segmentation results were corroborated by using visual inspection of LGE CMR brightness levels and one endocardial bipolar voltage map. The measured extent of pre-ablation fibrosis fell within the normal range for the specific arrhythmia phenotype. 3D volume renderings of segmented post-ablation enhancement emulated the expected ablation lesion patterns. By comparing our technique with other related approaches that proposed different threshold levels (although they also relied on reference regions from within the LABP) for segmenting enhancement in LGE CMR data-sets of AF patients, we illustrated that the cut-off levels employed by other centres may not be usable for clinical studies performed in our centre.

**Conclusions:**

The proposed technique has great potential for successful employment in the AF management within our centre. It provides a highly desirable validation of the LGE CMR technique for AF studies. Inter-centre differences in the CMR acquisition protocol and image analysis strategy inevitably impede the selection of a universally optimal algorithm for segmentation of enhancement in AF studies.

## Background

### Clinical backdrop

Atrial fibrillation (AF) occurs when chaotic electrical activity develops in the atrial walls, causing the atrial muscle cells to contract irregularly and rapidly. Apart from electrical and contractile remodelling, it is well established [1, 2] that AF is also associated with structural remodelling, including left atrial fibrosis. Fibrotic changes in the left atrial substrate have been postulated [1, 2] to underlie the persistence and sustainability of AF. AF is the most common heart rhythm disorder; it affects 2 % of the population, a figure that is rising fast [3]. AF has been connected [4–6] with a notable reduction in quality of life, poor mental health, disability, and a significant increase in the risk of stroke, dementia, and death. It is a serious and growing drain on the purse of healthcare providers worldwide, with the estimated [3] annual (2005) cost of AF treatment in USA to be $6.65 billion.

Ablation (by catheter or surgical techniques) of the left atrium (LA) has been widely accepted [7] as a clinical therapy for AF patients that are refractory to anti-arrhythmic medicines and direct current cardio-version. It is based on the principle of restoring sinus rhythm by forming lesions (scar tissue) that either electrically isolate the triggers of ectopic beats or modify the AF-favouring left atrial substrate. Despite efforts to improve targeting and delivery of AF ablation, the long-term durable restoration of sinus rhythm is achieved only for a moderate part of the AF population. That is to say, AF-free rates after a single ablation vary between 30 and 50 % at 5 years follow-up [8, 9]. Patients who fail the initial ablation commonly undergo redo procedures, thus reducing the cost-effectiveness of AF ablation and increasing the risk of associated complications.

The high failure rate of ablation in AF patients is in part attributable to: (1) Difficulty in identifying befitting ablation candidates. Excluding patients unlikely to benefit from AF ablation would improve the success rates. (2) Incapability to establish the ideal ablation strategy for every patient. Experts have agreed [10] that one ablation strategy does not fit all AF patients. (3) Limited information about the location and extent of the ablation-induced injury during and/or after the procedure. Such information could be used to identify gaps in ablation lines and guide initial/redo operations. (4) Restricted knowledge of the lesion set permanence at the procedure time. This renders the ablation end-point definition dubious. Silent electrograms in the ablated area are frequently temporary. Indeed, restored electrical connection at previously targeted sites almost invariably takes place in patients who return for a repeat procedure [10, 11].

The advent of late gadolinium (Gd) enhancement cardiovascular magnetic resonance (LGE CMR) more than a decade ago allowed the differentiation between normal and diseased left ventricular myocardium [12]. Its foundation lies in the slow washout kinetics of Gd agents in abnormal tissue. In brief, the delayed removal of Gd from abnormal cardiac tissue areas results in them being detectable as enhanced (i.e., brighter than healthy myocardium) zones. More recently, three-dimensional (3D) high spatial resolution LGE CMR has shown promise in increasing the success rates of AF ablation procedure. It offers the potential to deal with the AF ablation failing causes described above by non-invasively imaging left atrial (1) gradual native fibrosis associated with AF [13–30], (2) iatrogenic ablation-induced lesion sets [11, 15, 16, 18, 22, 26, 27, 31–47]. The former LGE CMR-derived tissue characterization has been suggested to (1) assess patient suitability for AF ablation by identifying potential non-responders [13–16, 18, 19, 23], (2) define the most appropriate ablation approach [15, 16, 21], (3) select anticoagulation strategy [16–18]. Correspondingly, the latter LGE CMR-derived tissue visualization has been proposed to (1) guide redo ablation procedures [16, 18, 22, 32, 36, 47], (2) deter the inadequate/ample lesion formation in the real-time (i.e., time-of-procedure) setting by assisting in the definition of a punctual ablation endpoint [16, 38–40, 42–44]. The use of LGE CMR to define native left atrial fibrosis associated with AF has been corroborated by histopathology studies [23, 48]. Similarly, a comprehensive histological validation of using LGE CMR to characterize AF ablation-induced wall injury has been presented [49].

### Challenges and current situation in the enhancement segmentation literature

The potential role of LGE CMR in the AF management discussed in the previous section underlines the great necessity for accurate enhanced tissue segmentation. However, this segmentation is challenged by many factors. At first, the wall of the LA is very thin. This, when combined with the current limits of CMR spatial resolution, results in the contrast between healthy (non-enhanced) and abnormal (enhanced) left atrial tissue being poorly visualized. Simultaneously, the mean intensity of enhanced regions varies (with the complex response to scan parameters and patient physiology) in unpredictable ways rendering the selection of a signal intensity threshold not so straightforward. In addition, there are few enhanced structures in the proximity of the LA (such as the mitral valve leaflets, the ascending and descending aorta walls, the right atrial wall at the septum, etc.) that are not related to left atrial fibrosis or ablation lesion and need to be meticulously distinguished. Likewise, false positives may be also generated by the navigator beam [50], which, in turn, reduces LGE CMR’s ability to visualize abnormal cardiac tissue near the two right pulmonary veins of the LA. To add to the above, the typically irregular heart-rate of AF patients results [51] in (1) ghosting artefacts that further degrade image quality, (2) failure to accentuate the existent abnormality due to imperfect healthy myocardium nulling. Despite the difficulties in classifying enhanced tissue in the LA, several studies have proposed related techniques. Next, an overview of the previously published approaches is provided.

To begin with, few AF studies [32, 41, 44] have performed manual segmentation of enhancement by relying on visual perception of experts. However, such an approach is considerably time-consuming and labor-intensive. In addition, it is very difficult to train new technicians to perform this task. Besides, the manual enhancement classification is prone to high intra- and inter-observer variability (even among experts) due to the factors (discussed above) that challenge the enhancement classification and also due to the high degree of patchiness that is characteristic of LGE CMR data-sets of AF patients. As a result, the manual segmentation of enhancement in AF is regarded as a rather unfavourable approach for use in clinical practice.

Motivated by earlier LGE CMR studies [52] on the infarcted left ventricle, a common recipe for segmenting left atrial enhanced tissue in AF patients is using a fixed (i.e., the same for every AF patient) signal intensity threshold expressed in terms of the two basic statistical measures [namely mean value and standard deviation (SD)] of the signal intensity distribution within a reference non-enhanced (healthy) region. With regard to pre-ablation LGE CMR, example reported intensity threshold levels above which a left atrial wall voxel was defined as fibrotic tissue are: (1) the three [18] SDs above the mean intensity of a region of the left atrial blood-pool (LABP), (2) the four [19] SDs above the mean intensity of a region of the LABP, (3) the two [22] SDs above the mean intensity of a healthy left atrial wall region, and (4) the six [27] SDs above the mean intensity of a region of the left ventricular wall. Analogously, example threshold levels that have been used in post-ablation LGE CMR studies to distinguish between healthy and abnormal tissue are the three [18] SDs above the mean intensity of a region of the LABP, the six [38, 42, 53] SDs above the mean intensity of a region of the right ventricular wall, the two [22] and three [11, 15, 33, 35, 36, 45] SDs above the mean intensity of a healthy left atrial wall region, and the six [27] SDs above the mean intensity of a region of the left ventricular wall. At the same time, other fixed approaches have proposed using 40 % [47] and 50 % [27] of the maximum left atrial wall intensity as post- and pre-ablation enhancement thresholds, respectively, and also the 50 % [27] of the maximum mitral valve intensity as a cut-off level for both pre- and post-ablation enhanced tissue. However, one downside of all these techniques is that they require segmentation of an additional reference region.

To further compensate for the measurement variability due to inter-patient differences (such as body mass index, hematocrit, glomerular filtration rate, etc.), several studies have suggested [13–17, 30, 46, 48, 54] to employ a varying (among different patients) threshold level that is benchmarked against the statistics of a healthy region. However, these methods are impeded by lack of automation since the threshold selection for every patient is always decided interactively by an experienced observer. An alternative empirical method [34, 37] proposed to use the minimum intensity that eliminated most of the blood-pool pixels in order to acquire a patient-specific enhancement threshold. As well as lack of objectivity and automation, one would expect such an approach to suffer from poor reproducibility all the same. More recently, a method [20] using graph-cuts was brought forward. According to this technique the enhancement segmentation is expressed as a Markov random field energy function minimization problem. However, this method involves a computationally demanding iterative process where hundreds of thousands nodes require processing. As a result, the graph-cuts method does not lend itself well for the real-time direct visualization of left atrial wall tissue destruction.

To summarize, the current situation in the literature is that the computer-aided classification of enhanced tissue in LGE CMR of AF is still an open question, and no algorithm has been deemed clearly better than others [55]. To add to the above, the number of centres that have reported successful application of LGE CMR to guide clinical AF strategies remains low, while the debate on this technique’s diagnostic ability for AF still holds [24, 41, 53, 56].

In this paper, we propose a technique to automatically segment enhanced tissue within the left atrial wall of (pre-ablation and 3 months post-ablation) LGE CMR data-sets of long-standing persistent AF patients studied at our centre. We employ thresholds that are benchmarked against the statistics of the whole LABP. The test-set cross-validation mechanism is applied to determine the input feature representation and algorithm that best predict enhancement threshold levels. The proposed enhancement classification algorithm was designed to provide the following advantages: (1) Self-regulated classification without requiring expert user interaction, (2) simplicity to implement allowing fast availability of results, (3) freedom from intra- and inter-observer variability, (4) provision of reproducible results, (5) lack of need to manually outline an additional healthy myocardial region, (6) development was specific to the LA, and (7) production of realistic estimates regardless mean enhancement intensity and image contrast ratio.

## Methods

### Study design

Thirteen patients (9 male, 62 ± 11 years old) presented to Royal Brompton Hospital for first-time ablation to treat long-standing persistent drug-refractory AF. 3D LGE CMR data-sets acquired both pre-procedurally and at 3 months post-ablation were included in this study. We addressed the problem of automatically segmenting left atrial wall enhanced tissue from these data-sets. The image quality was assessed by an expert in CMR. The study was approved by the local (UK) research ethics committee. Written informed consent was obtained from all research participants.

### The ablation procedure

All ablation procedures were performed under general anesthesia. Thoracoscopic bipolar radiofrequency surgical ablation was performed on five consecutive patients. The pulmonary veins were isolated using a clamp and a posterior wall box lesion was created using linear ablation connecting the two superior and inferior veins. The left atrial appendage was excluded in three patients. Percutaneous catheter ablation was delivered on the remaining patients, who underwent a stepwise lesion set strategy: (1) Antral pulmonary vein isolation, (2) linear ablation at the left atrial roof and mitral isthmus, and (3) ablation of the left atrial complex fractionated electrograms. Atrial anatomy was reconstructed with the NavX mapping system with an AFocusII catheter (St. Jude Medical, St. Paul, MN, USA). Radiofrequency ablation was performed with a 3.5-mm irrigated-tip catheter (ThermoCool, Biosense Webster, Diamond Bar, CA, USA).

### LGE CMR acquisition protocol

CMR was carried out using a Siemens Magnetom Avanto 1.5 Tesla scanner (Siemens Medical Systems, Erlangen, Germany). Imaging was performed 15 min after Gd administration (Gadovist—gadobutrol, 0.1 mmol/kg body weight, Bayer-Schering, Berlin, Germany) when a transient steady-state of Gd wash-in and wash-out of normal myocardium had been reached [57]. Transverse navigator-gated 3D LGE imaging was performed using a segmented gradient echo sequence as follows: 32–36 slices at 1.5 mm × 1.5 mm × 4 mm, reconstructed to 64–72 slices at 0.7 mm × 0.7 mm × 2 mm, generalised auto-calibrating partially parallel acquisition (GRAPPA) ×2, acquisition window 125 ms positioned within the subject-specific rest period, single R-wave gating, chemical shift fat suppression, centric *kz* and centric *ky* ordering, flip angle 20°, crossed-pairs navigator positioned over the dome of the right hemi-diaphragm with nominal navigator acceptance window size of 5 mm. The inversion time (TI) used for conventional two-dimensional (2D) breath-hold LGE imaging acquired with alternate R-wave gating was reduced for single R-wave gating by an amount depending on the patient’s heart rate. Nominal acquisition durations were 144–160 cardiac cycles, assuming 100 % respiratory efficiency.

### The proposed enhancement classification technique

In this section, we describe the work-flow for achieving automatic segmentation of enhancement in LGE CMR data-sets of AF patients studied at our center. In order to assess how well the proposed segmentation technique will generalize in independent (i.e., other than those included in this study) LGE CMR data-sets, we employed the test set cross-validation mechanism [58]. This avenue involved (1) learning the segmentation technique by relying only on a random selection of 3/4 of the study population (training LGE CMR data-sets), and (2) predicting the responses (enhancement distributions) of the remaining population sub-set (testing LGE CMR data-sets). To improve reliability of future estimations, three rounds of the cross-validation tool were performed.

In pursuance of classifying and isolating AF ablation-related injured tissue and/or pre-existent fibrotic tissue within the left atrial wall, the whole LABP was employed as the reference region. The reason for this choice is that the boundaries of the LABP chamber coincide with the left atrial endocardial surface. Therefore, no extra segmentation step (apart from the left atrial wall segmentation) was required to obtain the specific reference region. Following this selection, intensity *I* at each left atrial wall voxel *i* was normalized as

1 $$ NI\left( i \right) = \left[ {I\left( i \right){-}\mu_{\text{bp}} } \right]/\sigma_{\text{bp}} $$

where *NI* is the normalized intensity and *μ*_bp_ and *σ*_bp_ are the mean value and the standard deviation of the signal intensity distribution of the voxels that constitute the LABP.

Next, and with the view to acquiring the ‘‘actual’’ left atrial wall normalized intensity thresholds that mark out the lower boundaries of fibrotic and/or lesion tissue for the ‘‘training’’ data-sets of this study, we relied on an expert observer judgement-based approach. In particular, each of three specialists in LGE CMR was independently shown enhanced tissue classification results (super-imposed on the LGE CMR data-sets) for normalized intensity cut-off levels that varied from one to six, in steps of 1/8. Subsequently, each expert selected (based on visual perception) the most appropriate threshold level to define enhanced tissue for each data-set (patient). Our objective was to resolve whether a global threshold of normalized intensity would be appropriate for all patients, and if not, to determine the input feature representation and algorithm that best predict the output (i.e., threshold level for enhancement).

The segmentation of the left atrial wall from the LGE CMR data-sets is an integral part of the proposed classification of each left atrial wall voxel as healthy tissue versus fibrotic/injured tissue. In this study, the left atrial wall segmentation (that encompasses the LABP segmentation) was performed semi-automatically. At first, a user-guided level set-based 3D geodesic active contour method [59] was employed to demarcate the left atrial endocardial surface (which coincides with the boundaries of the LABP chamber). This automatic step was subsequently followed by an expert observer manual delineation of the left atrial wall epicardial surface. While performing this non-automatic step, extra care was taken to exclude nearby enhanced structures (such as mitral valve leaflets, aorta walls, and navigator-induced artefacts) not related to left atrial fibrosis or ablation lesion. To account for the intra- and inter-operator variability in this step (due to anatomic variability and partial volume effects), it was arranged to employ a consensus decision-making process. That is to say, the finalized version of every segmented left atrial wall was collaboratively generated by a group of three specialists in CMR of the LA that convened for this purpose. The whole left atrial wall segmentation was carried out using a free open-source segmentation software (ITK-SNAP) [60].

### Analyses

Initially, we aimed at qualitatively evaluating the accuracy of the learnt enhancement segmentation rule on the first-seen (testing) data-sets, by overlaying the enhanced tissue segmentation results upon the original LGE CMR transverse plane slices.

Due to the fact that the segmentation of enhancement in LGE CMR data-sets of AF patients is a complicated task that poses many challenges, the visual assessment of the results based on brightness levels is not alone sufficient. At the same time, both left atrial fibrosis and ablation injury (at 3 months post-ablation) have been shown [49, 61–63] to predict an impairment of atrial conduction/electric activation at the microscopic level. Therefore, there should be a correspondence between the highly current-resistant fibrotic deposits or ablation lesion sets (as these two are visualized by LGE CMR) and regions of low myocardial voltage. In the light of the above and with the view to improving the soundness of the results of this study, we set out to corroborate the proposed LGE CMR enhancement classification technique (apart from using visual inspection criteria based on brightness levels) also by using the unique endocardial bipolar voltage map that was available for one patient of this study and was acquired by a minimally-invasive electro-anatomic mapping (EAM) system. As well as they allow for the indirect assessment of left atrial substrate through voltage tissue characterization, EAM systems [64] also make provision for 3D cardiac chamber reconstruction, accurate navigation in the LA, assessment of adequate energy delivery etc. For this reason, EAM systems are regarded as the linchpin of modern complex AF ablations, and are routinely employed by ~90 % of the centres [10].

In addition, we also sought to rate the proposed enhancement segmentation technique: (1) In terms of the measured extent of the left atrial wall structural remodeling (fibrosis). (2) By looking into the suggested method’s capacity for reflecting ablation lesion patterns. To this end, we used 3D volume renderings of the classified enhancement superimposed on the segmented LABP to test the hypothesis that our enhancement segmentation technique (when it is applied to 3 months post-ablation LGE CMR data-sets) can recreate the (aimed) ablation lesions in a faithful way.

Finally, we investigated how the various threshold levels proposed by other AF studies [18, 19] (that also relied on reference regions from within the LABP) fare in LGE CMR data-sets acquired at our center.

## Results

Four pre-ablation LGE CMR data-sets were excluded by the CMR expert due to poor or non-diagnostic image quality. With regard to the automatic endocardial surface segmentation step, manual editing of the result of the active contour evolution was performed wherever there was a need.

The enhancement normalized intensity thresholds that were selected by the expert observers during the training for pre-ablation and 3 months post-ablation LGE CMR data-sets are given in Tables 1 and 2, respectively. Given the minor fluctuation in the outputs (selected thresholds) that was observed among patients and experts, it was decided that the input feature space has dimension zero and global thresholds of *T*_*PRE*_ = 1 1/4 (for pre-ablation LGE CMR data-sets) and *T*_*POST*_ = 1 5/8 (for data-sets acquired at 3 months post-ablation) best predict enhanced tissue for AF patients studied at out center. The three cross-validation rounds produced similar results. Therefore, these thresholds levels were used for all further analyses.

**Table 1:** Pre-ablation threshold levels selected by expert observers

	Observer 1	Observer 2	Observer 3
Patient 1	1 1/4	1 1/4	1 1/4
Patient 2	1 1/8	1 1/4	1 1/8
Patient 3	1 1/4	1 1/4	1 1/4
Patient 4	1 1/4	1 1/4	1 3/8
Patient 5	1 1/4	1 1/4	1 1/4
Patient 6	1 1/4	1 3/8	1 3/8
Patient 7	1 1/4	1 1/4	1 1/4

The normalized intensity levels that were selected by the expert observers to mark out the lower boundary of enhanced tissue in the training (*N* = 7) pre-ablation LGE CMR data-sets. Possible threshold levels ranged from 1 to 6 in increments of 1/8

**Table 2:** 3 months post-ablation threshold levels selected by expert observers

	Observer 1	Observer 2	Observer 3
Patient 1	1 5/8	1 5/8	1 5/8
Patient 2	1 5/8	1 5/8	1 5/8
Patient 3	1 4/8	1 5/8	1 4/8
Patient 4	1 5/8	1 5/8	1 5/8
Patient 5	1 6/8	1 5/8	1 5/8
Patient 6	1 5/8	1 5/8	1 5/8
Patient 7	1 5/8	1 5/8	1 5/8
Patient 8	1 5/8	1 5/8	1 5/8
Patient 9	1 5/8	1 6/8	1 6/8
Patient 10	1 5/8	1 5/8	1 5/8

The normalized intensity levels that were selected by the expert observers to mark out the lower boundary of enhanced tissue in the training (*N* = 10) 3 months post-ablation LGE CMR data-sets. Possible threshold levels ranged from 1 to 6 in increments of 1/8

**Fig. 1:**
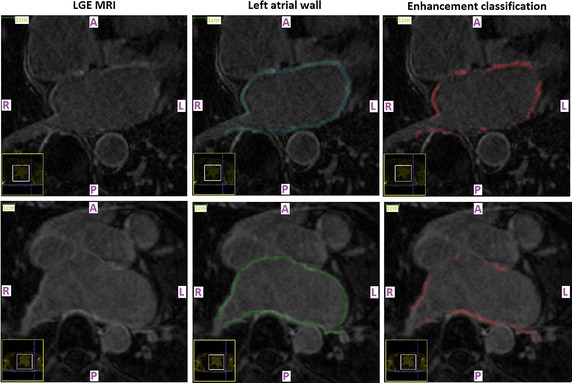
Pre-ablation segmentation results. Enhancement segmentation results on testing baseline LGE CMR data-sets of two randomly selected long-standing persistent AF patients (*top* patient 1, *bottom* patient 2). *Left* Short-axis slices of the original data-sets zoomed-in on the left atrium. *Middle* Segmented left atrial walls. The masks appear highlighted and have been super-imposed on the LGE CMR slices. *Right* The enhancement segmentation results of the proposed algorithm. Results appear highlighted in *red* and have been super-imposed on the LGE CMR slices. *A* anterior, *P* posterior, *L* left, *R* right

**Fig. 2:**
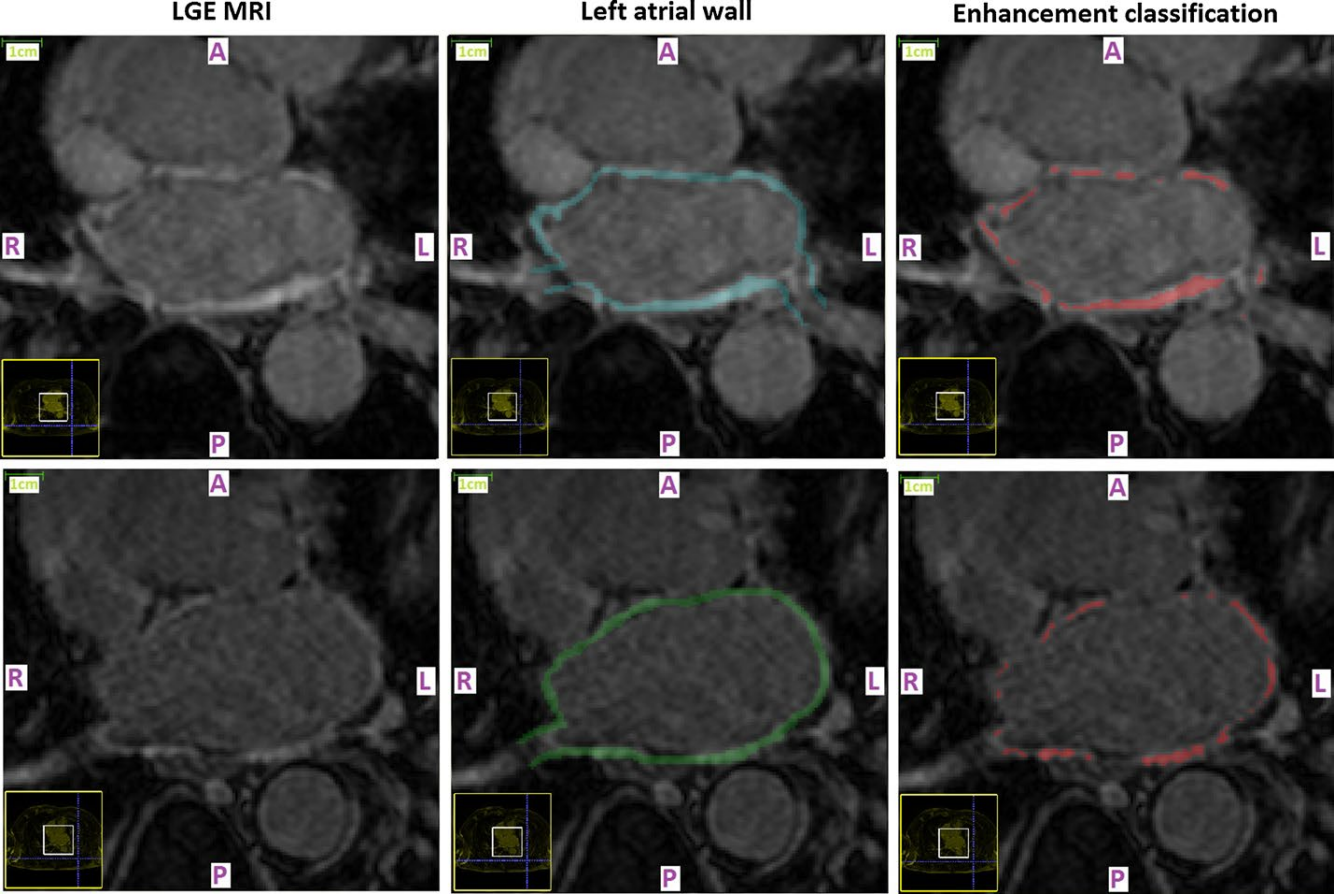
Post-ablation segmentation results. Enhancement segmentation results on testing 3 months post-ablation baseline LGE CMR data-sets of two randomly selected long-standing persistent AF patients (*top* patient 1, *bottom* patient 2). *Left* Short-axis slices of the original data-sets zoomed-in on the left atrium. *Middle* Segmented left atrial walls. The masks appear highlighted and have been super-imposed on the LGE CMR slices. *Right* The enhancement segmentation results of the proposed algorithm. Results appear highlighted in *red* and have been super-imposed on the LGE CMR slices. *A* anterior, *P* posterior, *L* left, *R* right

A qualitative evaluation of the predictive power of the chosen thresholds on first-seen (testing) LGE CMR data-sets is provided in Figs. 1 and 2, where the enhanced tissue segmentation results have been overlaid upon the original transverse plane slices of ‘‘testing’’ LGE CMR data-sets. It was generally observed that the proposed thresholds resulted in enhancement classifications that are in good concordance with visual perception for both pre-ablation (baseline) and 3 months post-ablation data-sets.

**Fig. 3:**
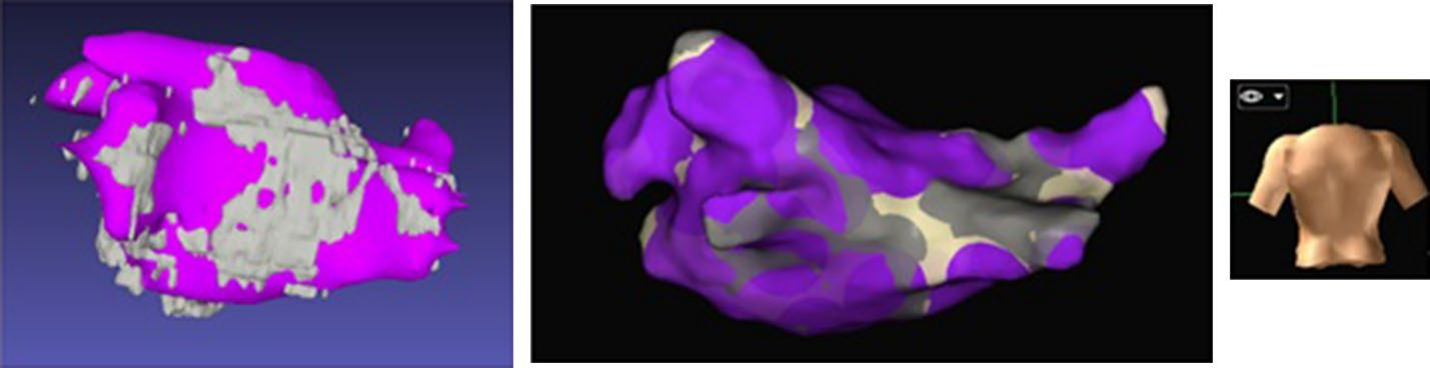
CMR versus invasive electro-physiology. Juxtaposition of the left atrial wall tissue classified as ‘‘pre-existent fibrosis’’ [*left*, in *gray*—obtained from a baseline LGE CMR scan and projected onto the segmented left atrial blood-pool (LABP), shown in *pink*] and the registered bipolar voltage map [*middle*—measured with a *NavX* EAM system: healthy left atrial wall tissue is showing in *purple*, while *gray* represents low voltage tissue]. The body illustration in the right-hand side image serves to clarify that the other two images refer to posteroanterior (PA) views of the left atrium (LA). CMR scar map compares well to the corresponding endocardial bipolar voltage map

An additional substantiation of our segmentation method against the unique endocardial bipolar voltage map that was available for one patient of this study is demonstrated in Fig. 3. A visually appreciable correlation between left atrial regions of classified fibrotic deposit (in a pre-ablation LGE CMR) and areas of low (i.e., <0.5 mV) bipolar endocardial voltage can be seen.

**Fig. 4:**
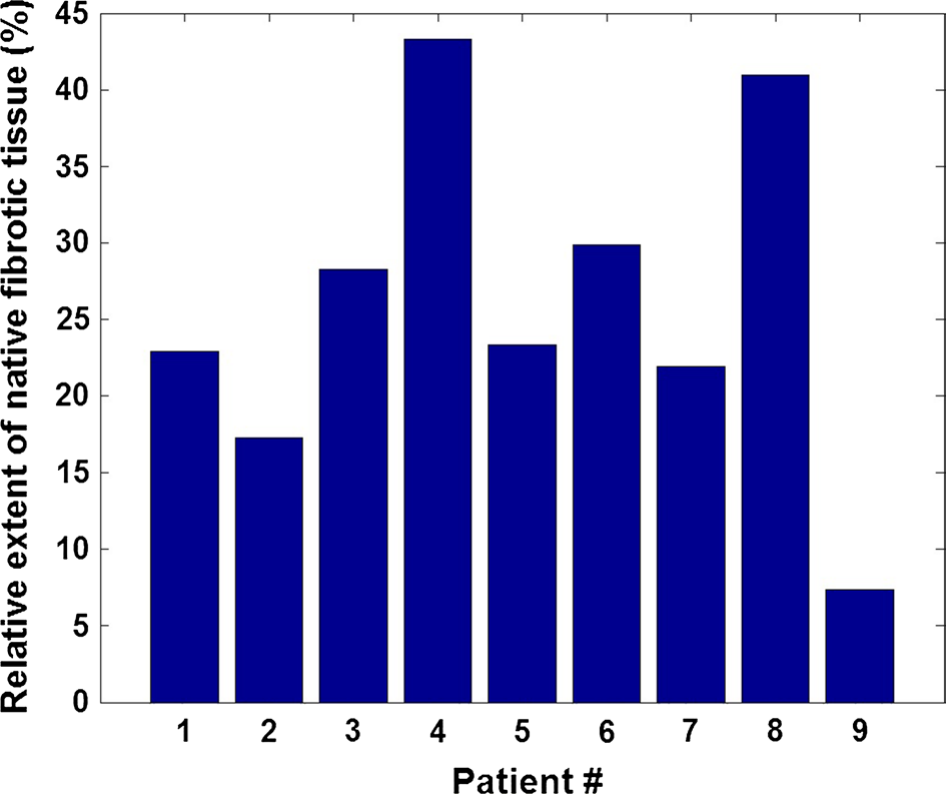
The relative extent of native fibrosis. The relative extent of native fibrosis as measured from the baseline LGE CMR data-sets of the long-standing persistent AF patients of this study. Fibrotic tissue extent is expressed as a percentage of the overall left atrial wall volume

By surveying the baseline scans of this study, we found that the mean relative extent of the measured native fibrosis associated with AF was 26.14 ± 11.17 % (Fig. 4; Table 3). This figure falls within the expected [13] range for the specific arrhythmia phenotype.

**Table 3:** Group statistics of the relative extent of native fibrosis

	Mean	Standard deviation
Relative extent of native fibrosis (%)	26.14	11.17

The mean and standard deviation of the relative extent of native fibrosis, summarizing the measurements from the baseline LGE CMR data-sets of the long-standing persistent AF patients of this study. Fibrotic tissue extent is expressed as a percentage of the overall left atrial wall volume

**Fig. 5:**
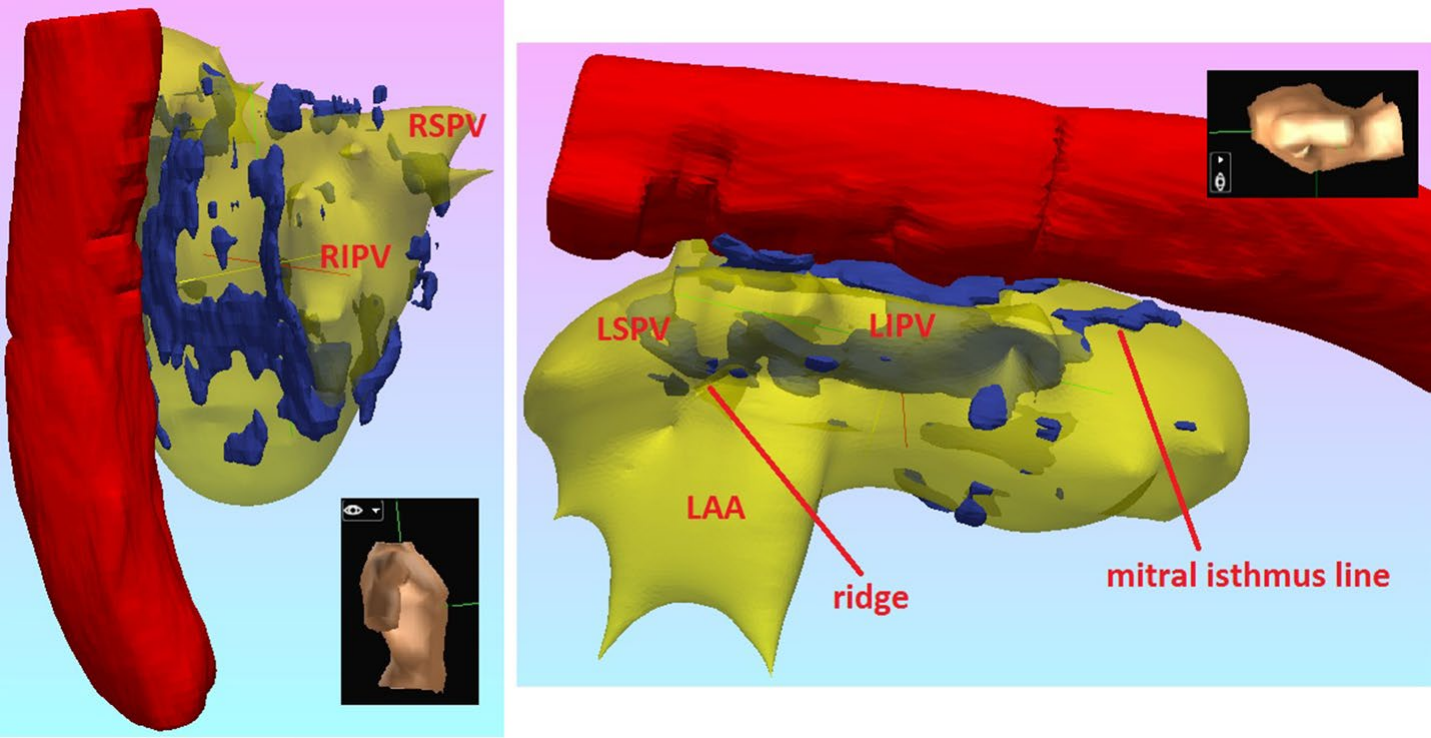
3D volume renderings of the ablation lesions. 3D volume renderings of the classified enhancement (in *blue*—obtained from a 3 months post-ablation LGE CMR data-set) overlaid upon the segmented and 3D rendered left atrial blood-pool (LABP, in semi-transparent *yellow*). The segmented and 3D rendered aorta is also displayed in *red*. The left-hand side image is the right view of the left atrium (LA) when the body is in vertical position, while the right-hand side image is the left view of the LA when the body is in prone position. Enhancement distribution comprised encirclement of each ipsilateral pair of pulmonary veins at their antra, as well as a linear pattern at the mitral isthmus, as might be expected by the respective ablation techniques. *RSPV* right superior pulmonary vein, *RIPV* right inferior pulmonary vein, *LSPV* left superior pulmonary vein, *LIPV* left inferior pulmonary vein, *LAA* left atrial appendage

The 3D visualization of the 3 months post-ablation LGE CMR data-sets of this study showed that the revealed distributions of classified enhancement typically emulated the ablation lesion patterns (acquired after implementing the ablation strategies described in “The ablation procedure”). A representative example is shown in Fig. 5, where (the 3D volume rendering of) the classified enhancement distribution comprises encirclement of each ipsilateral pair of pulmonary veins at their antra, as well as a linear pattern at the mitral isthmus.

As an interim summary, the results presented up to this point allowed us to reach the decision that any left atrial wall voxel *i* with normalized intensity *NI*(*i*) above *T*_*PRE*_ = 1 1/4 in a baseline scan is defined as abnormal native fibrotic tissue, whereas any left atrial wall voxel *i* with normalized intensity *NI*(*i*) above *T*_*POST*_ = 1 5/8 in a 3 months post-ablation scan is classified as abnormal native fibrotic or AF ablation-related injured tissue.

**Fig. 6:**
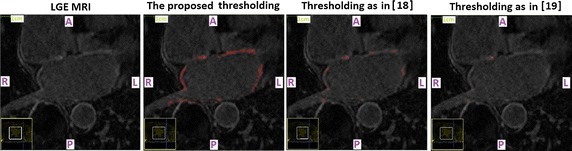
Comparison with threshold levels proposed by other studies on pre-ablation data-sets. Comparison of enhancement segmentation techniques on a base-line LGE CMR data-set of a long-standing persistent AF patient. *Left* Short-axis slices of the original data-sets zoomed-in on the left atrium. *Second from left* The enhancement segmentation results of the algorithm proposed in this study. *Second from right* The enhancement segmentation results of the algorithm proposed in [18]. *Right* The enhancement segmentation results of the algorithm proposed in [19]. All enhanced tissue segmentation results appear highlighted in *red* and have been super-imposed on the LGE CMR slices. The threshold levels proposed in other studies (*T*_*PRE*_ = 3 for study cited in [18], and *T*_*PRE*_ = 4 for study cited in [19]) led to gross underestimations of the enhancement. *A* anterior, *P* posterior, *L* left, *R* right

**Fig. 7:**
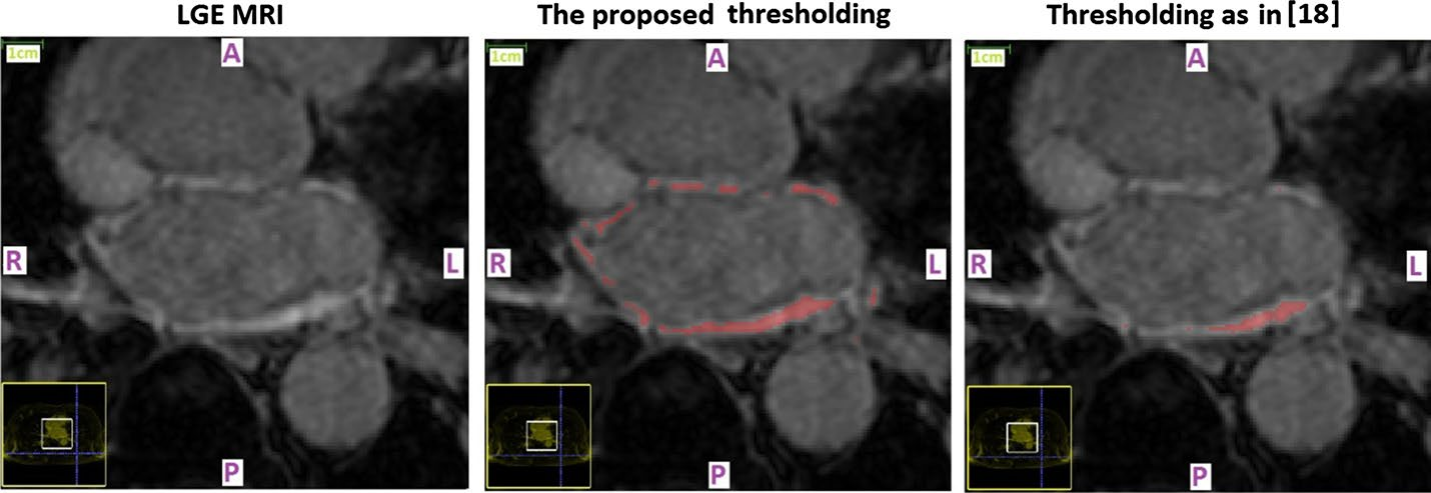
Comparison with threshold levels proposed by other studies on 3 months post-ablation data-sets. Comparison of enhancement segmentation techniques on a 3 months post-ablation LGE CMR data-set of a long-standing persistent AF patient. *Left* Short-axis slices of the original data-sets zoomed-in on the left atrium. *Middle* The enhancement segmentation results of the algorithm proposed in this study. *Right* The enhancement segmentation results of the algorithm proposed in [18]. All enhanced tissue segmentation results appear highlighted in *red* and have been super-imposed on the LGE CMR slices. The threshold level proposed in another study (*T*_*POST*_ = 3 for study cited in [18]) led to gross underestimation of the enhancement. *A* anterior, *P* posterior, *L* left, *R* right

Finally, comparisons of the proposed enhancement segmentation technique with other related papers [18, 19] that suggested using different threshold levels for segmenting enhancement in LGE CMR data-sets of AF patients are shown in Figs. 6 and 7. The figures illustrate that, when it comes to LGE CMR data-sets acquired to our centre, our proposed thresholds conspicuously outperform those proposed in studies cited in [18, 19].

## Discussion

### The main contribution

AF represents a major public health problem. Putting this abnormal heart condition in the disease rates comparison context, today everyone aged forty or over has a lifetime risk of developing AF of at least one in four, compared, for example, with one in eight for breast cancer in women of the same age group [65, 66]. On top of this, the AF prevalence is expected [3] to double by 2050. Ablation treatment of AF is associated with modest outcomes. In order for LGE CMR to ameliorate the AF management, the ready availability of the accurate enhancement segmentation is required. However, the computer-aided classification of enhanced tissue in LGE CMR of AF is still an open question. This study was designed to retrospectively propose a fast automatic method that reliably distinguishes enhanced (abnormal) from non-enhanced (healthy) tissue within the left atrial wall of (baseline and 3 months post-ablation) LGE CMR data-sets of long-standing persistent AF patients studied at our centre. Detection and isolation of enhancement was achieved by employing thresholds benchmarked against the statistics of the entire LABP. On the whole, global normalized intensity threshold levels *T*_*PRE*_ = 1 1/4 and *T*_*POST*_ = 1 5/8 were found to segment enhancement in data-sets acquired pre-ablation and at 3 months post-ablation, respectively. The proposed segmentation algorithm combines the following advantages: (1) It is self-regulated without requiring expert user interaction, (2) it is simple to implement, and runs in a couple of seconds on a typical personal computer (PC) allowing fast availability of results, (3) it eliminates observer bias, (4) it provides reproducible results, (5) it does not require manual outlining of an additional healthy myocardial region, (6) it has been developed particularly for the LA, (7) it produces realistic estimates regardless mean enhancement intensity and image contrast ratio.

The predictive power of the proposed enhancement segmentation rule was verified on first-seen (testing) LGE CMR data-sets through the cross-validation mechanism. This tool allows for minimally biased results (by preserving the total blinding of the testing data-sets from the training procedure), while at the same time uses the available data as effectively as possible. Apart from corroborating the segmentation results by relying on visual inspection of LGE CMR brightness levels, we also verified the proposed technique by using the unique endocardial bipolar voltage map that was available for one patient of this study. The observation of a marked correspondence between the fibrotic deposits (classified by our segmentation technique) and regions of low myocardial voltage (measured with an EAM system) boosts further the employment of non-invasive LGE CMR in clinical practice as a guidance tool on left atrial substrate characterization. In addition, by measuring the extent of classified fibrosis and correlating it with the studied arrhythmia phenotype, we showed that the proposed segmentation technique favours the settlement of the link between the degree of fibrosis and the disease severity in AF [13]. This type of measurement is also key for noninvasively identifying patients that are unlikely to benefit from ablation. Furthermore, the suggested method’s capacity for reflecting the expected ablation lesion patterns was demonstrated by using 3D volume rendering techniques. Such knowledge could prove useful in pinpointing ablation line gaps and guiding repeat operations. All in all, the proposed segmentation technique: (1) has great potential for successful employment in the AF management within our centre, and (2) provides a highly desirable (according to the present status of the related literature) validation of the LGE CMR technique for AF studies.

### Global versus patient-specific thresholding

LGE CMR data-sets from AF patients are highly variable with respect to image noise, contrast, and mean enhancement intensity. To address this problem, some studies [20] have suggested employing a patient-specific (rather than a fixed) threshold level. However, we found in this study that the selected fixed threshold levels applied well to all LGE CMR data-sets regardless image contrast ratio and mean enhancement intensity. A possible explanation for this is that the normalization of the left atrial wall intensities (against the statistics of the signal intensity distribution of the voxels that constitute the LABP in the same data-set) appears to have compensated for the contrast and mean enhancement intensity variability.

### Enhancement segmentation: post-ablation as opposed to pre-ablation data-sets

In this study, there was a sense of greater confidence in the segmentation results of post-ablation rather than baseline LGE CMR data-sets. This is in accordance with the surrounding circumstances since the native pre-existent fibrosis is known [26] to have a more diffuse distribution, as opposed to the iatrogenic scarring which exhibits a focal pattern. However, the 3 months post-ablation LGE CMR data-sets of AF patients are expected to involve enhancement that corresponds to both native fibrotic tissue and radiofrequency energy application lesion sets. It is our opinion that at the moment it is very challenging to distinguish between these two types of enhancement that reside on a post-ablation data-set, despite the fact that the pre-procedural data-sets are also available for these patients. The reasons for this are (1) the inaccessible/unpredictable intensity scaling (that takes place within the MR scanner before exporting the data) which results in the two types of enhancement having arbitrary and possibly common intensities, and (2) the two types of enhancement are contiguous. Finally, it only makes things worse the fact that these two sources of enhancement sit at the opposite ends of a ring diameter and snarl at each other, since AF-related fibrosis has been postulated to be a central component of arrhythmia maintenance, while, on the other hand, ablation-related scar aims at favouring freedom of arrhythmia.

### Left atrial wall segmentation

Segmentation of the left atrial wall is a crucial step before segmenting enhancement. The consensus manual delineation of the left atrial wall epicardial border is a downside of this study, as it is a time-consuming step taking up to 1 h. Our decision to implement this step manually was based on the fact that fully automatic approaches at the moment result in unreliable left atrial wall segmentations and, as a result, they require significant manual feedback. In any case, this paper is concerned only with the classification of enhancement within the left atrial wall of AF patients; the determination of the left atrial wall boundaries is beyond the scope of this study. Even though our proposed enhancement classification technique was applied to left atrial walls that had been segmented semi-automatically, it could also be executed with no modification within fully automatically segmented left atrial walls.

### Comparison with other papers

**Fig. 8:**
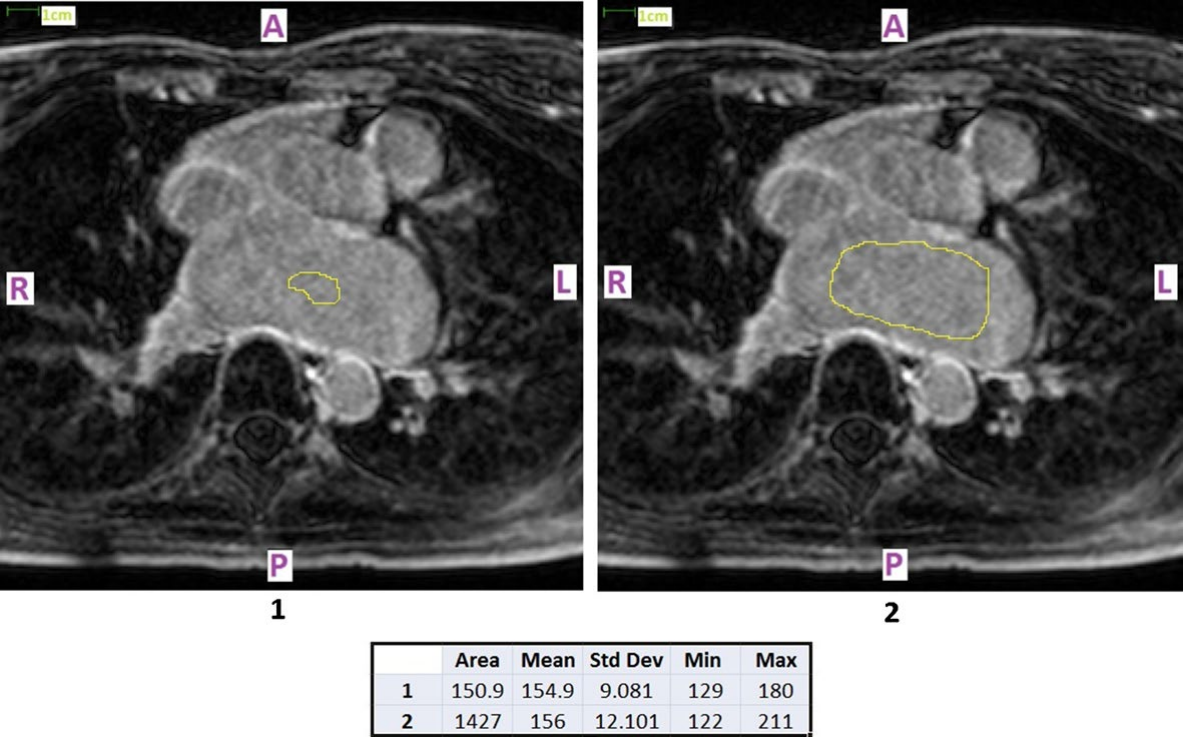
Regional variation in the reference region statistics. The borders of the reference region within the left atrial blood-pool (LABP) are drawn in *yellow*. By choosing a larger area, the standard deviation of the signal intensity distribution also increased

Another contribution of this paper is that we compare our technique with other related approaches that proposed different threshold levels (although they also relied on reference regions from within the LABP) for segmenting enhancement in LGE CMR data-sets of AF patients. The failure of threshold levels suggested by other papers was illustrated for data-sets acquired in our centre. A reason for this discrepancy may be the inter-centre variability in CMR acquisition parameters, such as the optimal timing of imaging after contrast administration, choice and ideal dosage of contrast agent, selection of the best TI etc. Another possibility is that the differences in the proposed thresholds arose out of regional variation in the reference region statistics. In particular, whereas this study employed the whole LABP, segmented directly for the LGE CMR image, the other two papers segmented the LABP from the 3D magnetic resonance angiography (MRA) sequence, and then registered this segmentation with the LGE CMR image. Finally, to account for the inherent spatial error (that occurred in the process of registering the non-electrocardiography-gated MRA sequence with the electrocardiography and respiratory motion gated free-breathing LGE sequence), the authors of [18, 19] concluded their reference region by performing mathematical morphology shrinkage. Therefore, their reference region was most likely a smaller sub-region within the most central part of the LABP which (in our LGE CMR data-sets) was observed (Fig. 8) to have significantly different statistic measures (standard deviation) from the entire LABP.

To summarize, threshold levels employed by other centres may not be usable for clinical studies performed in our centre; instead, the operator would have to resort to threshold re-adjustment in order to achieve accurate assessment of left atrial substrate, and prevent unnecessary ablation. Inter-centre differences in the CMR acquisition protocol and image analysis strategy inevitably impede the selection of a universally optimal algorithm for segmentation of enhancement in AF studies.

### Other limitations of this study

The major limitation of this study was the relatively small sample size. Therefore, further larger studies are required to confirm these results. Furthermore, only one voltage map was available for validation. Another limitation of this paper is that few of the criteria used to assess the proposed technique are qualitative, mainly due to the lack of a reliable gold standard ground truth. While qualitative criteria have been rich in communicating the message, it is recognized, at the same time, that quantitative tools would have allowed for much greater precision and objectiveness in comparisons.

## Conclusions

We proposed a method to distinguish enhanced (abnormal) from non-enhanced (healthy) tissue within the left atrial wall of (pre-ablation and 3 months post-ablation) LGE CMR data-sets from long-standing persistent AF patients studied at our centre. Segmentation of enhancement was achieved by employing thresholds benchmarked against the statistics of the whole LABP. Global normalized intensity threshold levels *T*_*PRE*_ = 1 1/4 and *T*_*POST*_ = 1 5/8 were found to segment enhancement in data-sets acquired pre-ablation and at 3 months post-ablation, respectively. The proposed segmentation algorithm combines the following advantages: (1) It is self-regulated, (2) it is simple to implement allowing fast availability of results, (3) it eliminates observer bias, (4) it provides reproducible results, (5) it does not require manual outlining of an additional healthy myocardial region, (6) it has been developed particularly for the left atrium, (7) it produces realistic estimates regardless mean enhancement intensity and image contrast ratio. The segmentation results were corroborated by relying on visual inspection of brightness levels of LGE CMR images and one endocardial bipolar voltage map. The measured extent of pre-ablation fibrosis fell within the normal range for the specific arrhythmia phenotype. 3D volume renderings of segmented post-ablation enhancement emulated the expected ablation lesion patterns. The proposed technique has great potential for successful employment in the AF management within our centre. The results provide a highly desirable (according to the present status of the related literature) validation of the LGE CMR technique for AF studies. The cut-off levels employed by other centres may not be usable for clinical studies performed in our centre. Inter-centre differences in the CMR acquisition protocol and image analysis strategy inevitably impede the selection of a universally optimal algorithm for segmentation of enhancement in AF studies. Further larger studies are required to confirm the results of this paper.

## Abbreviations

2D: two-dimensional
3D: three-dimensional
A: anterior
AF: atrial fibrillation
CMR: cardiovascular magnetic resonance
EAM: electro-anatomic mapping
GRAPPA: generalised auto-calibrating partially parallel acquisition
L: left
LA: left atrium
LAA: left atrial appendage
LABP: left atrial blood-pool
LGE: late gadolinium enhancement
LIPV: left inferior pulmonary vein
LSPV: left superior pulmonary vein
MRA: magnetic resonance angiography
P: posterior
PA: postero-anterior
PC: personal computer
R: right
RIPV: right inferior pulmonary vein
RSPV: right superior pulmonary vein
SD: standard deviation
TI: inversion time
